# Peripheral Nitrosative Stress in Pediatric Anxiety Disorders: Elevated Serum Nitric Oxide Levels

**DOI:** 10.5152/eurasianjmed.2026.251235

**Published:** 2026-06-15

**Authors:** Merve Okuyucu, Mehmet Fatih Ceylan, Selma Tural Hesapçıoğlu, Seda Kafalı, Salim Neşelioğlu, Özcan Erel

**Affiliations:** 1 Department of Child and Adolescent Psychiatry, Prof. Dr. Cemil Taşçıoğlu City Hospital, İstanbul, Türkiye; 2 Department of Child and Adolescent Psychiatry, Ankara Yıldırım Beyazıt University Faculty of Medicine, Ankara, Türkiye; 3 Department of Child and Adolescent Psychiatry, Kırıkkale Yuksek İhtisas Hospital, Kırıkkale, Türkiye; 4 Department of Biochemistry, Ankara Yıldırım Beyazıt University Faculty of Medicine, Ankara, Türkiye

**Keywords:** Anxiety disorders, biomarkers, child, nitric oxide, oxidative stress, reactive nitrogen species

## Abstract

**Background:**

The developing pediatric brain is vulnerable to nitrosative stress. This study investigated serum nitric oxide (NO), inducible nitric oxide synthase (iNOS), and S-nitrosothiols (SNOs) as peripheral biomarkers in children with anxiety disorders (ADs) and their associations with symptom severity.

**Methods:**

Eighty-one participants aged 7-17 years were enrolled, including 40 drug-naive participants with ADs and 41 age- and sex-matched healthy controls (HCs). Diagnoses were established using the Schedule for Affective Disorders and Schizophrenia for School-Age Children—Present and Lifetime Version, DSM-5—Turkish version (K-SADS-PL-DSM-5-T). Symptom severity was assessed using the Screen for Child Anxiety Related Emotional Disorders (SCARED) and the Clinical Global Impression–Severity (CGI-S) Scale. Data were analyzed using between-group comparisons, correlation analyses, covariance analyses (MANCOVA/ANCOVA), controlling for age, sex, and body mass index (BMI), and receiver operating characteristic (ROC) analysis.

**Results:**

Serum NO levels were significantly higher in the AD group compared with HCs (*P* = .028), whereas iNOS and SNO levels did not differ (*P *> .05). In adjusted analyses controlling for age, sex, and BMI, NO remained significantly higher, and iNOS and SNO levels were also significantly higher in the AD group. No marker (NO, iNOS, SNOs) correlated significantly with anxiety severity (all *P* > .05). Receiver operating characteristic analysis indicated modest diagnostic accuracy for NO (area under the curve (AUC) = 0.629, 95% CI: 0.506-0.752, *P* = .046), while iNOS and SNOs were not significant.

**Conclusion:**

Elevated NO, alongside adjusted increases in iNOS and SNO, supports altered peripheral nitrosative signaling in pediatric ADs. Nitric oxide showed modest discriminative value.

**Main Points**

- Nitrosative stress has been implicated in the pathophysiology of psychiatric disorders, yet, evidence in children remains limited.
- This study demonstrated significantly higher serum nitric oxide (NO) levels in children and adolescents with anxiety disorders (AD) compared to healthy controls.
- In initial unadjusted analyses, there was no difference in inducible nitric oxide synthase (iNOS) and S-nitrosothiol (SNO) levels between groups. However, when adjusting for factors such as age, sex, and BMI, the AD group showed increased levels of iNOS and SNO.
- Elevated NO may reflect early peripheral nitrosative dysregulation in pediatric anxiety disorders.
- The findings suggest that peripheral NO could serve as a potential biomarker for early anxiety vulnerability in youth.

## Introduction

Anxiety disorders (ADs) are among the most common psychiatric conditions in children, impacting about 5% of the population of youth.^1^ Anxiety disorders typically emerge during school age and can persist into adolescence and adulthood, affecting academic, social, and emotional domains.^2^^,^^3^ Despite their frequent occurrence, chronic course, and functional impairment that affects daily life, the neurobiological mechanisms of pediatric ADs remain insufficiently clarified.^2^

Oxidative and nitrosative stress processes, reflecting redox imbalance in the developing brain, can impair neuronal signaling through overproduction of reactive oxygen and nitrogen species, impair synaptic plasticity, and promote neuroinflammation.^4^^-^^6^ Nitrosative pathways are closely linked to oxidative processes; both reflect disruptions in redox balance.^7^ In particular, the high metabolic activity and underdeveloped antioxidant systems of the pediatric brain may create a significant susceptibility to such dysregulation.^6^ Meta-analytic findings from diverse childhood psychiatric disorders support the broader relevance of redox mechanisms to pediatric psychopathology.^8^^-^^10^ While direct evidence in pediatric ADs remains limited,^11^ findings from related psychiatric conditions suggest that redox dysregulation may represent a peripheral window into neurobiological vulnerability underlying anxiety pathophysiology.^12^^,^^13^

Among redox-related pathways, nitric oxide (NO) and its derivatives—such as inducible nitric oxide synthase (iNOS) and S-nitrosothiols (SNOs)—play key roles in cellular signaling and stress response.^4^^-^^6^ As a biomarker, serum NO levels are considered an index of overall nitrergic activity, while iNOS reflects chronic and stress-related NO production, and SNOs are considered indicators of cumulative nitrosative modifications, reflecting long-term molecular signatures of NO signaling.^5^^,^^14^ Together, NO, iNOS, and SNO may provide complementary information regarding nitrosative signaling. Aberrations in these markers have been implicated in several psychiatric and neurological disorders,^5^^,^^12^^,^^13^^,^^15^^,^^16^ suggesting their potential as peripheral indicators of disrupted redox homeostasis relevant to emotional and stress-related psychopathology.^12^^,^^15^

By comparison, nitrosative mechanisms have received far less attention in children. One cross-sectional study has examined these pathways, reporting no differences in serum NO and adrenomedullin between children with ADHD, ADs, and healthy controls (HCs).^17^ In contrast, other pediatric reports demonstrated elevated NO and 3-nitrotyrosine levels in autism with bipolar comorbidity,^18^ and significantly increased NO levels in children diagnosed with stuttering.^19^ These limited and heterogeneous findings highlight the need for further studies assessing nitrosative biomarkers to clarify their relevance in pediatric ADs. Importantly, no prior study has examined NO, iNOS, and SNO simultaneously in pediatric ADs, which may help clarify whether nitrosative dysregulation is a peripheral marker of early-onset ADs.

It was hypothesized that children diagnosed with ADs would show increased serum levels of NO, iNOS, and SNOs relative to HCs. These biomarkers were also expected to be related to anxiety severity and to distinguish children with ADs from HCs.

## Material and Methods

### Participants

This cross-sectional study was carried out at the outpatient clinic specializing in child and adolescent psychiatry of Ankara Yıldırım Beyazıt University Yenimahalle Training and Research Hospital between February 2021 and February 2022. Ethical approval was obtained from the Ankara City Hospital Ethics Committee (No: 1682, Date: 06.01.2021), and the study was conducted in accordance with the Declaration of Helsinki (2000 revision). Written informed consent was obtained from the legal guardians, and verbal assent was obtained from all participating children and adolescents.

A priori power analysis was performed using G*Power (version 3.1.9.7),^20^ which indicated that a minimum of 25 participants per group was required (*α* = 0.05, power = 0.95). The final sample included 40 children with ADs and 41 age- and sex-matched HCs, ensuring sufficient statistical power.

Participants in both groups were required to be between 7 and 17 years of age and to provide written informed consent from their legal guardians, together with verbal assent from the participants. For the AD group, additional criteria included a first-time clinical diagnosis of AD, absence of psychiatric comorbidities other than ADs, and no prior use of psychotropic medication. For the HC group, eligibility required the absence of any current or past psychiatric disorder and no history of psychotropic medication use.

Exclusion criteria for both groups included: 1) age below 7 or above 17 years; 2) presence of chronic diseases (such as metabolic, genetic, endocrinological, neurological disorders, or anemia); 3) intellectual disability was diagnosed clinically, based on developmental history, academic performance, and available educational/medical records; 4) history or current use of psychotropic medications; 5) current use of any treatments or supplements; 6) smoking, alcohol, or drug use; 7) presence of an active infection within the preceding 2 weeks; and 8) body mass index (BMI) not within the age- and sex-specific normal range.^21^ The presence of chronic medical conditions was evaluated through clinical interviews with parents and participants and by reviewing available electronic medical records.

**Figure 1.**
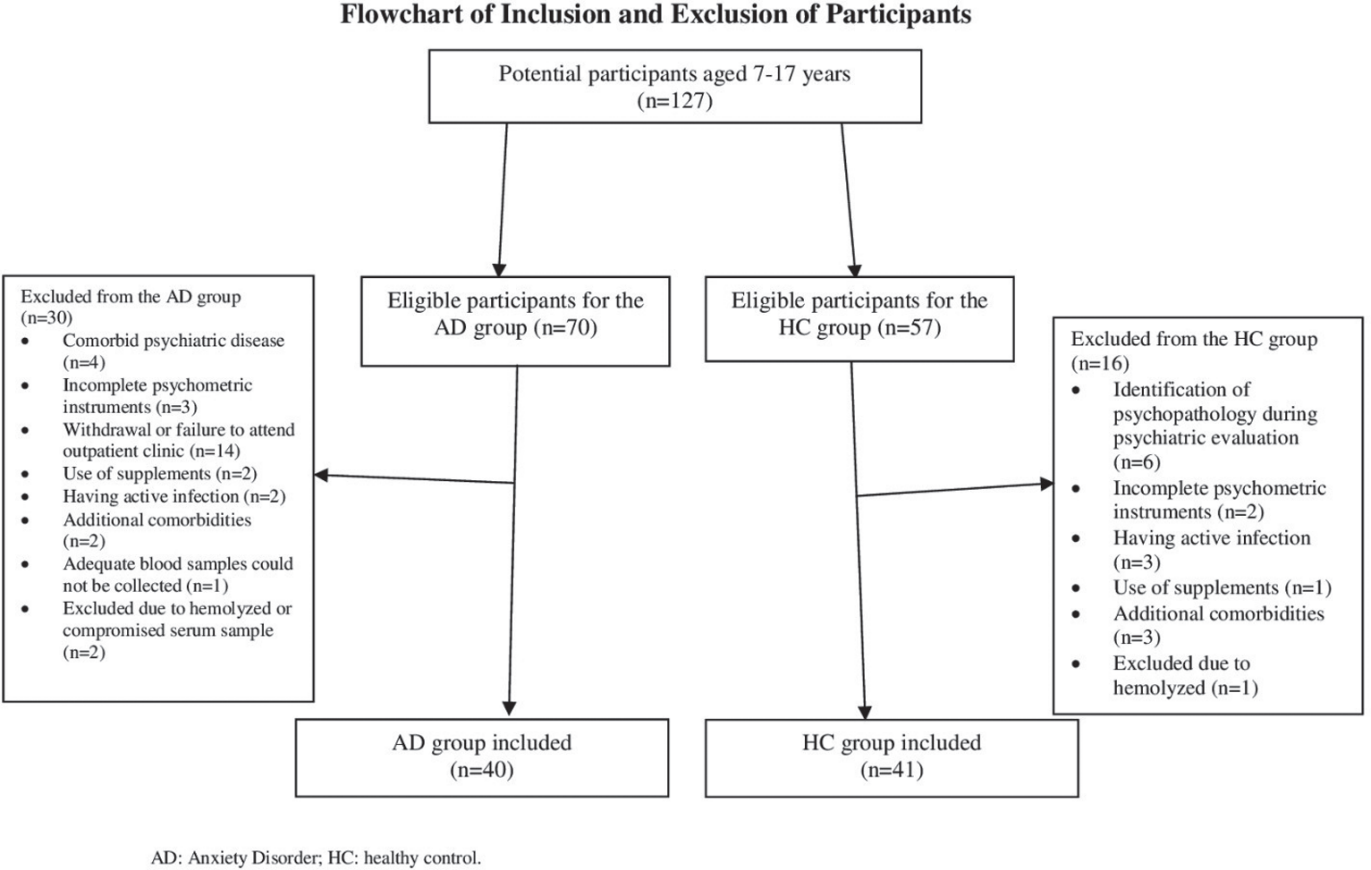
Flowchart of inclusion and exclusion of participants.

Initially, 70 children with ADs and 57 HCs were recruited to participate. See Figure 1 for the participant selection flowchart.

### Diagnostic Assessment

Children in the AD group were diagnosed with ADs based on the criteria of the Diagnostic and Statistical Manual of Mental Disorders, Fifth Edition (DSM-5*)*.^22^ The HC group consisted of children assessed in the child and adolescent psychiatry outpatient clinic and confirmed to have no psychiatric disorders.

**Figure 2.**
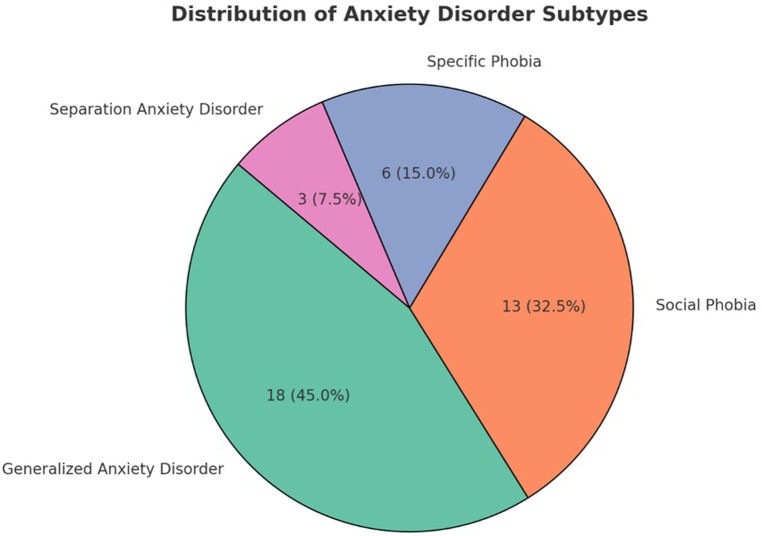
Distribution of anxiety disorders subtypes.

Psychiatric diagnoses were assessed using the Schedule for Affective Disorders and Schizophrenia for School-Age Children-Present and Lifetime Version (K-SADS-PL-DSM-5-T),^23^ which has been validated in Turkish and shown to be an effective tool for diagnosing major childhood psychiatric disorders.^24^ All evaluations were conducted by clinicians under the supervision of experienced child and adolescent psychiatrists and/or professors. Diagnoses were assigned according to DSM-5 criteria based on the K-SADS-PL-DSM-5-T interview. Children were diagnosed with either a single or multiple ADs. Among the 40 children with ADs, 27 had a single diagnosis, 11 had 2 co-occurring ADs, and 2 had 3 ADs; no child had more than 3. Overall, 13 children exhibited at least 1 comorbid AD. The most common comorbid diagnoses were generalized anxiety disorder (n = 8) and social anxiety disorder (n = 5), followed by specific phobia (n = 1) and separation anxiety disorder (n = 1). See Figure 2 for the distribution of primary AD subtypes.

This study used ChatGPT to improve language during the preparation of the manuscript. However, all content of the study has been reviewed and edited by the authors following the use of AI/LLM tools.

### Measurements

Following the psychiatric evaluation, sociodemographic and clinical information was collected through a structured questionnaire completed by clinicians based on interviews with participants and their guardians. Symptom severity was rated by the researchers using the Clinical Global Impression Scale-Severity (CGI-S),^25^ while anxiety symptoms were assessed with the Screen for Child Anxiety Related Emotional Disorders (SCARED) self-report scale^26^ completed by the participants. Participants’ socioeconomic level was categorized as low, middle, or high according to the national income distribution and the official minimum wage levels in Türkiye during 2021-2022.^27^ A certified dietitian obtained anthropometric measurements (height and weight) to ensure accurate BMI calculation.

### Sociodemographic Data Form

Sociodemographic and clinical data of the participants were collected utilizing a study-specific form developed by the researchers, completed by the clinician during the clinical interview based on information provided by the parents.

### Screen for Child Anxiety Related Emotional Disorders

The SCARED^26^ is a 41-item self-report scale used to assess a broad range of anxiety symptoms in children. Each item is scored on a 3-point Likert-type scale (0-2), resulting in a total score ranging from 0 to 82, with higher scores indicating more severe symptoms. In its Turkish adaptation, the study on validity and reliability found the internal consistency coefficient (Cronbach’s α) for the total scale to be 0.88.^28^

### Clinical Global Impression Scale-Severity

The Clinical Global Impression Scale (CGI) is a widely used clinician-rated scale to evaluate illness severity, overall improvement, and treatment response. The severity subscale (CGI-S) is a 7-point Likert scale, scored from 1 (not at all ill) to 7 (extremely ill),^25^ and was utilized in this study to assess each participant’s overall clinical severity.

### Biochemical Analyses

Blood samples were obtained after overnight fasting, centrifuged at 3000 rpm for 10 minutes to isolate serum, then aliquoted and stored at –80°C until analysis. When fasting status could not be ensured, blood collection was postponed to the following morning. Serum NO, iNOS, and SNO concentrations were quantified using standardized spectrophotometric and enzyme-linked immunosorbent assay (ELISA)-based assays.

Nitric oxide was measured colorimetrically via the Griess reaction, iNOS using a human ELISA kit, and SNO via the Saville colorimetric method.^29^ All assays were conducted in duplicate, with intra- and inter-assay coefficients of variation maintained below 10%. Results were expressed as µmol/L for NO and SNO, and pg/mL for iNOS.

### Statistical Analysis

Statistical analyses were conducted utilizing IBM SPSS Statistics for Windows, version 26.0 (IBM Corp., Armonk, NY, USA). The normality of data was evaluated using the Shapiro–Wilk test. Between-group differences were evaluated with independent-samples *t* tests or Mann–Whitney *U* tests, as appropriate, and categorical variables were compared using Pearson’s chi-square (*χ*^2^) test. Additionally, a multivariate analysis of covariance (MANCOVA), followed by univariate analyses of covariance (ANCOVA), was performed to compare biomarker levels between groups while controlling for potential confounding variables (age, sex, and BMI). The examination of adjusted group differences and estimated marginal means was conducted, and a Bonferroni correction was implemented for pairwise comparisons. Effect size estimation used Cohen’s d for continuous variables, while Phi (φ) coefficients were applied to categorical variables. According to Cohen’s conventions, *d* values of 0.2, 0.5, and 0.8 were considered small, medium, and large, respectively; for φ, thresholds of 0.1, 0.3, and 0.5 were used. For MANCOVA and ANCOVA analyses, effect sizes were reported as partial eta squared (partial η^2^). The relationships between symptom severity (SCARED and CGI-S scores) and biomarker levels were analyzed using Spearman’s rank correlation. Receiver operating characteristic (ROC) analyses were performed to assess the diagnostic efficacy of serum NO, iNOS, and SNO levels in differentiating AD cases from HCs. The area under the curve (AUC) and its corresponding 95% CI were reported for each biomarker. The Youden index (J = sensitivity + specificity − 1) was used to identify the optimal cut-off point. Sensitivity, specificity, as well as positive and negative predictive values (PPV and NPV), were then computed to evaluate diagnostic performance. Statistical analyses were performed using 2-tailed tests, with *P* < .05 indicating statistical significance.

## Results

### Sociodemographic and Clinical Characteristics

In the AD group, 9 participants were boys and 31 were girls; the HC group included 10 boys and 31 girls. The groups did not differ significantly in age, sex, height, or weight (*P > *.05). The median age was 14.5 years (range: 7-17) in the AD group and 12.0 years (range: 7-17) in the HC group. The SCARED scores were markedly higher in the AD group (*P < *.001*, r *= 0.86). A summary of demographic and clinical characteristics is provided in Table 1.

### Comparison of Nitrosative Stress Biomarkers Between Groups

As shown in Table 2, unadjusted analyses demonstrated significantly higher NO levels in the AD group compared with HCs (4.58 ± 2.40 vs 3.53 ± 1.76 µmol/L,* P* = .028), whereas iNOS and SNO levels did not differ significantly between groups (*P *> .05).

Prior to follow-up univariate analyses, a one-way MANCOVA was conducted with NO, iNOS, and SNO as dependent variables, group as the fixed factor, and age, sex, and BMI as covariates. The overall multivariate effect of group was significant (Wilks’ λ = 0.715, F(3,74) = 9.853, *P < .001*, partial η^2^ = 0.285).

Subsequent ANCOVA analyses controlling for age, sex, and BMI revealed significantly higher adjusted NO (F(1,76) = 4.556,* P =* .036, partial η^2^ = 0.057), iNOS (F(1,76) = 4.451, *P = *.038, partial η^2^ = 0.055), and SNO levels (F(1,76) = 7.162, *P = *.009, partial η^2^ = 0.086) in the AD group compared with HCs (Table 2).

### Correlation Between Nitrosative Stress Parameters and Clinical Symptom Severity in Children with Anxiety Disorders

Spearman correlation analyses revealed no significant associations between nitrosative stress markers (NO, iNOS, and SNO) and clinical symptom severity (SCARED and CGI-S scores), with all *P *> .05. For detailed results, see Table 3.

### Diagnostic Utility of Nitrosative Stress Markers

Receiver operating characteristic analyses were conducted to assess the discriminative ability of serum nitrosative stress markers (NO, iNOS, and SNO) in differentiating children with ADs from HCs.

**Figure 3.**
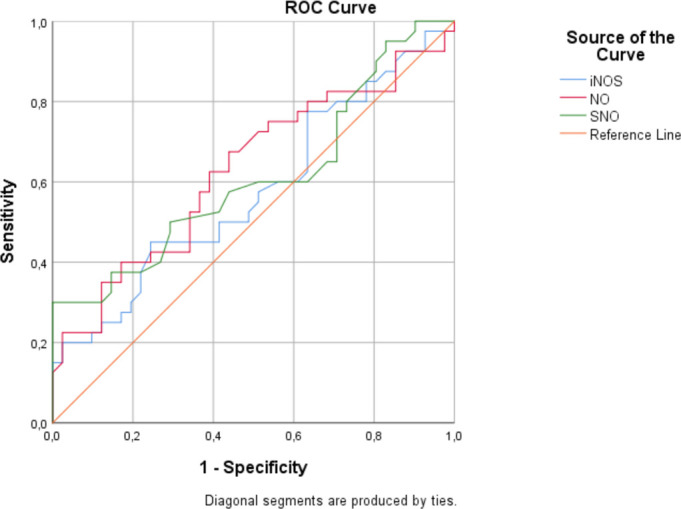
Comparative receiver operating characteristic curves for serum nitrosative stress markers

Nitric oxide levels demonstrated modest diagnostic accuracy, with an AUC of 0.629 (95% CI *=* 0.506-0.752, *P* = .046). The optimal cut-off value (3.66 µmol/L; Youden index, 0.24) yielded 67.5% sensitivity and 56.1% specificity, corresponding to a PPV of 60.0% and an NPV of 63.9% based on sample prevalence. Inducible nitric oxide synthase levels did not show statistically significant discriminative ability (AUC *= *0.577, 95% CI *= *0.451-0.702, *P* = .234). The optimal cut-off (1149.9 pg/mL; Youden index, 0.07) produced 70.0% sensitivity but low specificity (36.6%), indicating limited diagnostic value. Similarly, SNO levels did not demonstrate statistically significant discriminative ability (AUC *=* 0.609, 95% CI *= *0.485-0.734, *P* = .090). The best cut-off (0.63 µmol/L; Youden index, 0.14) provided 57.5% sensitivity and 56.1% specificity. Combined ROC curves are presented in Figure 3.

## Discussion

This study examined peripheral serum nitrosative stress markers in drug-naive children and adolescents with ADs compared with HCs. Nitric oxide levels were significantly higher in the AD group, while iNOS and SNO levels did not differ in unadjusted analyses but became significantly higher after adjustment for age, sex, and BMI. None of the measured markers showed significant associations with symptom severity. Although the discriminative performance of NO was modest, these findings may reflect alterations in peripheral nitrosative balance among children with ADs. To current knowledge, this is the first study to assess NO, iNOS, and SNOs simultaneously in a well-characterized, medication-naive, comorbidity-free pediatric AD sample, minimizing pharmacological and diagnostic confounders.

A key finding of the present study was the significantly higher serum NO levels observed in children with ADs than in HCs. Nitric oxide participates in several biological processes related to stress reactivity, neurovascular regulation, and synaptic signaling—mechanisms that are closely linked to anxiety-related neurobiology.^4^^,^^6^ Consistent with these biological roles, clinical studies have investigated alterations in NO metabolites in ADs. However, findings in adult populations have been inconsistent, with reports of both increased and decreased NO levels.^12^^,^^15^^,^^30^ These discrepancies may reflect differences in methodological approaches, illness chronicity, or treatment exposure. In pediatric populations, evidence remains limited. One prior study did not detect differences in serum NO levels between children with ADHD, ADs, and HCs.^17^ In contrast, the sample revealed elevated peripheral NO levels, suggesting that nitrosative alterations may also be involved in childhood-onset ADs.

An important finding of the study was that iNOS and SNO levels differed significantly between groups after adjusting for age, sex, and BMI. The emergence of group differences only after covariate adjustment suggests that developmental and metabolic factors may influence the expression of peripheral nitrosative markers. Higher iNOS levels in the AD group may reflect an increased capacity for sustained, high-output NO production. Unlike constitutive nitric oxide synthase (NOS) isoforms, iNOS can generate larger and more prolonged amounts of NO,^5^^,^^31^ which may favor cumulative nitrosative modifications and subtle disturbances in redox homeostasis. Additionally, differences in SNO levels suggest alterations in the regulation of protein S-nitrosylation, a reversible post-translational modification through which NO exerts many of its biological effects.^14^^,^^32^^,^^33^ These findings may therefore indicate a shift in peripheral nitrosative signaling in children with ADs, potentially reflecting developmental changes in redox-related stress regulation.

Anxiety disorders represent a heterogeneous diagnostic category encompassing multiple subtypes, including generalized anxiety disorder, social anxiety disorder, and specific phobias, which may differ in their biological stress responses.^2^ Differences in the distribution of AD subtypes across various studies may thus contribute to inconsistencies observed in research findings regarding stress-related peripheral biomarkers. By focusing on a pediatric population, the present study reduces potential confounding from illness chronicity and pharmacological treatment, enabling investigation of nitrosative alterations in pediatric ADs.

None of the serum markers (NO, iNOS, and SNO) showed significant associations with clinical severity. One possible explanation is that the limited symptom range in this newly diagnosed, drug-naive cohort may have restricted variability. In addition, peripheral NO levels may fluctuate on shorter timescales than subjective symptom reports. Increased NO may also reflect a compensatory rather than a pathogenic mechanism, potentially representing adaptive neurovascular or synaptic responses to early stress exposure.^4^^,^^6^ Developmental factors such as sex, pubertal status, and illness duration may further influence the relationship between nitrosative markers and symptom severity.

Although iNOS and SNO levels differed between groups after adjustment, these markers did not demonstrate significant discriminative ability in ROC analyses. In the present study, only NO showed significant discriminative ability in differentiating children with ADs from HCs. Specifically, NO demonstrated modest diagnostic accuracy with a sensitivity of 67.5% and a specificity of 56.1%. Although this level of discrimination is insufficient for independent diagnostic use, these findings suggest that NO may provide complementary biological information to support clinical assessment, particularly in cases with overlapping symptom presentations. This observation is in line with recent studies examining nitrosative stress markers as potential diagnostic indicators in other childhood psychiatric conditions.^18^^,^^19^ Because predictive values are prevalence-dependent, these diagnostic estimates may differ in clinical settings with lower AD prevalence.

### Limitations and Future Directions

Several limitations of this study should be acknowledged. First, information on pubertal status and hormone levels was not collected, which may influence nitrosative regulation. Moreover, peripheral biomarkers may not directly reflect central processes, and only 1 NOS isoform was quantified; future studies should include nNOS and eNOS. In addition, assessing symptom severity with subjective scales, along with the inclusion of physiological or neuroimaging measures, may strengthen the interpretation of biomarker associations. Furthermore, subgroup analyses regarding anxiety could not be conducted owing to the limited sample size. Finally, the duration of anxiety symptoms was not systematically assessed, which may be relevant when interpreting biomarker alterations in relation to illness chronicity. Longitudinal studies involving larger, diagnostically stratified, and sex-specific cohorts are necessary to elucidate the developmental trajectory of nitrosative dysregulation.

## Conclusions

Children and adolescents with ADs showed altered peripheral nitrosative profiles, including elevated NO and adjusted increases in iNOS and SNO. While the diagnostic accuracy of NO was modest, it may contribute to future biomarker models for early identification. Further studies are needed to clarify the role of nitrosative stress in pediatric ADs.

## Figures and Tables

**Table 1.** Sociodemographic and Clinical Characteristics of the Participants

Variables	HC Group (n = 41)	AD Group (n = 40)	χ^2^/t/z Value	P	Effect Size
Age (years), median (min-max)	12.0 (7-17)	14.5 (7-17)	Z = −1.889	.059	0.42
Gender (female/male, %)	31/10 (75.6/24.4)	31/9 (77.5/22.5)	χ^2^ = 0.040	.841	0.00
Height (cm)	148.63 ± 16.85	155.68 ± 15.95	t = 1.93	.057	0.43
Weight (kg)	44.0 (22.8-66.0)	51.9 (20.0-70.0)	Z = −1.602	.109	0.36
Socioeconomic level, n (%)					
Low	17 (41.5)	14 (35.0)	χ^2^ = 3.159	.206	0.20
Middle	11 (26.8)	18 (45.0)			
High	13 (31.7)	8 (20.0)			
CGI-S	–	6.0 (5-7)	–	–	–
SCARED	3.0 (0-13)	45.5 (37-73)	Z = −7.758	<.001	0.86

AD, anxiety disorder; CGI-S, Clinical Global Impression Scale-Severity; HC, healthy control; SCARED, screen for child anxiety–related emotional disorders.

**Table 2.** Comparison of Nitrosative Stress Parameters Between Groups

Biomarker	HC Group (n = 41)	AD Group (n = 40)	Unadjusted Test Statistic	Unadjusted P	Adjusted HC Mean (SE)	Adjusted AD Mean (SE)	ANCOVA F (1.76)	Adjusted P	Partial η^2^
NO	3.53 ± 1.76	4.58 ± 2.40	t = 2.243	.028	3.536 (0.337)	4.569 (0.341)	4.556	.036	0.057
iNOS	1310.51 ± 305.44	1441.72 ± 426.48	t = 1.595	.115	1294.187 (54.208)	1458.444 (54.894)	4.451	.038	0.055
SNO	0.62 (0.10-0.74)	0.65 (0.30-1.33)	Z = −1.697	.090	0.559 (0.030)	0.675 (0.031)	7.162	.009	0.086

Values are presented as mean ± SD for normally distributed variables and median (min–max) for non-normally distributed variables. Unadjusted comparisons were performed using Student’s t-test (NO, iNOS) or Mann–Whitney U-test (SNO). Adjusted means were estimated using ANCOVA controlling for age, sex, and body mass index (BMI).

AD, anxiety disorder; ANCOVA, analysis of covariance; HC, healthy control; iNOS, inducible nitric oxide synthase; NO, nitric oxide; SE, standard error; SNO, S-nitrosothiols.

**Table 3.** Spearman Correlation Analysis Between Nitrosative Stress Parameters and Clinical Symptom Severity

Parameters	SCARED (r/P)	CGI-S (r/P)
NO	−.042/.796	.083 / .609
iNOS	.047/.775	−.134 / .410
SNO	−.012/.942	−.016 / .922

CGI-S, Clinical Global Impression Scale-Severity; iNOS, inducible nitric oxide synthase; NO, nitric oxide; SCARED, screen for child anxiety related emotional disorders; SNO, S-nitrosothiols. No significant correlations were observed (P > .05).

## Data Availability

The data that support the findings of this study are available on request from the corresponding author.
